# gsufsort: constructing suffix arrays, LCP arrays and BWTs for string collections

**DOI:** 10.1186/s13015-020-00177-y

**Published:** 2020-09-22

**Authors:** Felipe A. Louza, Guilherme P. Telles, Simon Gog, Nicola Prezza, Giovanna Rosone

**Affiliations:** 1 Faculdade de Engenharia Elétrica, Universidade Federal de Uberlândia, Uberlândia, Brazil; 2 Instituto de Computação, Universidade Estadual de Campinas, Campinas, Brazil; 3 eBay Inc., San Jose, USA; 4 LUISS Guido Carli, University, Rome, Italy; 5 Dipartimento di Informatica, Università di Pisa, Pisa, Italy

**Keywords:** Suffix array, LCP array, Burrows–Wheeler transform, Document array, String collections

## Abstract

**Background:**

The construction of a suffix array for a collection of strings is a fundamental task in Bioinformatics and in many other applications that process strings. Related data structures, as the Longest Common Prefix array, the Burrows–Wheeler transform, and the document array, are often needed to accompany the suffix array to efficiently solve a wide variety of problems. While several algorithms have been proposed to construct the suffix array for a single string, less emphasis has been put on algorithms to construct suffix arrays for string collections.

**Result:**

In this paper we introduce gsufsort, an open source software for constructing the suffix array and related data indexing structures for a string collection with *N* symbols in *O*(*N*) time. Our tool is written in ANSI/C and is based on the algorithm gSACA-K (Louza et al. in Theor Comput Sci 678:22–39, 2017), the fastest algorithm to construct suffix arrays for string collections. The tool supports large fasta, fastq and text files with multiple strings as input. Experiments have shown very good performance on different types of strings.

**Conclusions:**

gsufsort is a fast, portable, and lightweight tool for constructing the suffix array and additional data structures for string collections.

## Background

The suffix array (${{\mathsf {S}}}{{\mathsf {A}}}$) [1] is one of the most important data structures in string processing. It enables efficient pattern searching in strings, as well as solving many other string problems [2–4]. More space-efficient solutions for such problems are possible by replacing the suffix array with an index based on the Burrows–Wheeler transform ($\mathsf {BWT}$) [5]. Many applications require additional data structures—most commonly, the longest common prefix ($\mathsf {LCP}$) [6] array and the document array (${{\mathsf {D}}}{{\mathsf {A}}}$) [7]—on top of ${{\mathsf {S}}}{{\mathsf {A}}}$ or $\mathsf {BWT}$. These structures, possibly stored in compressed form, serve as a basis for building modern compact full-text indices, which allow to efficiently pre-process and query strings in compact space.

There are several internal memory algorithms designed for constructing the suffix array and additional data structures when the input consists of a single string [8, 9]. While less emphasis has been put on specialized algorithms for string collections, in many applications the input is composed by many strings, and a common approach is concatenating all strings into a single one and using a standard construction algorithm. However, this approach may deteriorate either the theoretical bounds or the practical behavior of construction algorithms due to, respectively, the resulting alphabet size or unnecessary string comparisons [10–12].

Textual documents and webpages are examples of widespread large string collections. In Bioinformatics, important problems on collections of sequences may be solved rapidly with a small memory footprint using the aforementioned data structures, for example, finding suffix-prefix overlaps for sequence assembly [13], clustering cDNA sequences [14], finding repeats [15] and sequence matching [16].

In this paper we present gsufsort, an open source tool that takes a string collection as input, constructs its (generalized) suffix array and additional data structures, like the $\mathsf {BWT}$, the $\mathsf {LCP}$ array, and the ${{\mathsf {D}}}{{\mathsf {A}}}$, and writes them directly to disk. This way, applications that rely on such data structures may either read them from disk or may easily include gsufsort as a component. Large collections, with up to $2^{64}-d-2$ total letters in *d* strings, may be handled provided that there is enough memory. This tool is an extension of previous results [10], with new implementations of procedures to obtain the $\mathsf {BWT}$ and the generalized suffix array ($\mathsf {GSA}$) from ${{\mathsf {S}}}{{\mathsf {A}}}$ during output to disk, and with the implementation of a lightweight alternative to compute ${{\mathsf {D}}}{{\mathsf {A}}}$.

## Implementation

gsufsort is implemented in ANSI C and requires a single Make command to be compiled. It may receive a collection of strings in fasta, fastq or raw ASCII text formats and computes ${{\mathsf {S}}}{{\mathsf {A}}}$ and related data structures, according to input parameters. gsufsort optionally supports gzipped input data using zlib^1^ and kseq^2^ libraries. Setting command-line arguments allows selecting which data structures are computed and written on disk, and which construction algorithm is used (see below). Additionally, a function for loading pre-constructed data structures from disk is also provided.

Given a collection of *d* strings $$T^1, T^2, \dots , T^d$$ from an alphabet $$\Sigma =[1,\sigma ]$$ of ASCII symbols, having lengths $$n_1, n_2, \dots , n_d$$, the strings are concatenated into a single string $$T[0,N-1]=T^1\$ T^2\$ \cdots \$ T^d \$\#$$ using the same separator $ and an end-marker #, such that $ and # do not occur in any string $$T^i$$, and $$\#<$$ $ $$< \alpha$$ for any other symbol $$\alpha \in \Sigma$$. The total length of *T* is $$\sum _{i=1}^{d} (n_i +1) +1 = N$$.

Before giving details on gsufsort implementation, we briefly recall some data structures definitions. For a string *S* of length *n* let the suffix starting at position *i* be denoted $S_i$, $0\le i\le n-1$. The suffix array ${{\mathsf {S}}}{{\mathsf {A}}}$ of a string *S* of length *n* is an array with a permutation of $[0,n-1]$ that gives the suffixes of *S* in lexicographic order. The length of the longest common prefix of strings *R* and *S* is denoted by ${\mathsf {lcp}} (R,S)$. The $\mathsf {LCP}$ array for *S* gives the ${\mathsf {lcp}}$ between consecutive suffixes in the order of ${{\mathsf {S}}}{{\mathsf {A}}}$, that is $\mathsf {LCP} [0]=0$ and $\mathsf {LCP} [i]={\mathsf {lcp}} (S_{SA[i]},S_{SA[i-1]})$, $0< i\le n-1$. For a suffix array of a collection of strings, the position *i* of the document array ${{\mathsf {D}}}{{\mathsf {A}}}$ gives the string to which suffix $T_{{{\mathsf {S}}}{{\mathsf {A}}} [i]}$ belongs. For the last suffix $T_{N-1}=\#$ we have ${{\mathsf {D}}}{{\mathsf {A}}} [0]=d+1$. The generalized suffix array gives the order of the suffixes of every string in a collection, that is, the $\mathsf {GSA}$ is as an array of *N* pairs of integers (*a*, *b*) where each entry (*a*, *b*) represents the suffix $T^a_b$, with $1 \le a \le d$ and $0 \le b \le n_a-1$.

gsufsort uses algorithm gSACA-K [10] to construct ${{\mathsf {S}}}{{\mathsf {A}}}$ for the concatenated string $T[0,N-1]$, which breaks ties between equal suffixes from different strings $T^i$ and $T^j$ by their ranks, namely *i* and *j*. gSACA-K can also compute $\mathsf {LCP}$ and ${{\mathsf {D}}}{{\mathsf {A}}}$ during ${{\mathsf {S}}}{{\mathsf {A}}}$ construction, such that $\mathsf {LCP}$ values do not exceed separator symbols. gSACA-K runs in *O*(*N*) time using $O(\sigma )$ working space.

The $\mathsf {BWT}$ is calculated during the output to disk according to its well-known relation to ${{\mathsf {S}}}{{\mathsf {A}}}$ [3]

$$\begin{aligned} \mathsf {BWT} [i]=T[({{\mathsf {S}}}{{\mathsf {A}}} [i]-1) \bmod N]. \end{aligned}$$

The generalized suffix array ($\mathsf {GSA}$) can be computed by gsufsort from ${{\mathsf {S}}}{{\mathsf {A}}}$ and ${{\mathsf {D}}}{{\mathsf {A}}}$ during the output to disk, using the identity

1 $$\begin{aligned} \mathsf {GSA} [i] = {\left\{ \begin{array}{ll} ({{\mathsf {D}}}{{\mathsf {A}}} [i], {{\mathsf {S}}}{{\mathsf {A}}} [i]-{{\mathsf {S}}}{{\mathsf {A}}} [{{\mathsf {D}}}{{\mathsf {A}}} [i]]{-1}) &{} \text{ if } \, {{\mathsf {D}}}{{\mathsf {A}}} [i]>1 \\ ({{\mathsf {D}}}{{\mathsf {A}}} [i], {{\mathsf {S}}}{{\mathsf {A}}} [i]) &{} \text{ otherwise }. \end{array}\right. } \end{aligned}$$

We also provide a lightweight version (gsufsort-light) for the computation of ${{\mathsf {D}}}{{\mathsf {A}}}$. It uses less memory at the price of being slightly slower. It computes a bitvector ${\mathsf {B}} [0,N-1]$ with *O*(1) rank support [4] such that $B[i]=1$ if $T[i]=\$,$ and $B[i]=0$ otherwise. The values in ${{\mathsf {D}}}{{\mathsf {A}}}$ are obtained on-the-fly while ${{\mathsf {D}}}{{\mathsf {A}}}$ (or $\mathsf {GSA}$) is written to disk, through the identity

$$\begin{aligned} {{\mathsf {D}}}{{\mathsf {A}}} [i] = \mathsf {rank_1}({{\mathsf {S}}}{{\mathsf {A}}} [i])+1. \end{aligned}$$

## Results

We compared our tool and mkESA. mkESA [17] is a fast suffix array construction software designed for bioinformatics applications.

We ran both versions of our tool, gsufsort and gsufsort-light, to build arrays $\mathsf {GSA}$ and $\mathsf {LCP}$, while mkESA^3^ was run to build arrays ${{\mathsf {S}}}{{\mathsf {A}}}$ and $\mathsf {LCP}$ for the concatenation of all strings (using the same symbol as separators). The experiments were conducted on a single core of a machine with GNU/Linux (Debian 8, kernel 3.16.0-4, 64 bits) with an Intel Xeon E5-2630 2.40-GHz, 384 GB RAM and 13 TB SATA storage. The sources were compiled by GNU GCC version 4.8.4 with option -O3.

The collections we used in our experiments are described in Table 1. They comprise real DNAs, real proteins, documents, random DNA and random protein, and differ by their alphabet size and also by the maximum and average ${\mathsf {lcp}}$, which offer an approximation for suffix sorting difficulty.

**Table 1:** Collections

Collection	size	$\sigma$	N. of strings	Max. len.	Avg. len	Max. lcp	Avg. ${\mathsf {lcp}}$
shortreads	16.00	5	171.8	100	100	100	32.87
reads	16.00	6	57.3	300	300	300	91.29
pacbio	16.00	5	1.9	71,561	9117	3084	19.08
pacbio.1000	16.00	5	17.2	1,000	1000	876	18.67
uniprot	16.04	25	46.1	74,488	374	74,293	99.24
gutenberg	15.88	255	334.3	757,936	50	9060	18.97
random.dna	16.00	4	16.1	1,048,576	1,048,576	33	16.18
random.protein	16.00	25	16.1	1,048,576	1,048,576	13	6.89

Columns 2 and 3 show the collection size (in GB) and the alphabet size. Column 4 shows the number of strings (in millions). Columns 5 and 6 show the maximum and average lengths of strings in a collection. Columns 7 and 8 show the maximum and average ${\mathsf {lcp}}$ of strings in a collection

*Collections*

shortreads are Illumina reads from human genome trimmed to 100 nucleotides (http://ftp.sra.ebi.ac.uk/vol1/ERA015/ERA015743/srf);

reads are Illumina HiSeq 4000 paired-end RNA-seq reads from plant *Setaria viridis* trimmed to 300 nucleotides (http://www.trace.ncbi.nlm.nih.gov/Traces/sra/?run=ERR1942989);

pacbio are PacBio RS II reads from *Triticum aestivum* (wheat) genome (http://www.trace.ncbi.nlm.nih.gov/Traces/sra/?run=SRR5816161);

pacbio.1000 are strings from pacbio trimmed to length 1,000;

uniprot are protein sequences from TrEMBl dowloaded on May 28, 2019 (http://www.ebi.ac.uk/uniprot/download-center);

gutenberg are ASCII books in English from Project Gutenberg (http://www.gutenberg.org);

random-dna was generated with even sampling probability on the standard 4 letter alphabet;

random-protein was generated with even sampling probability on the IUPAC 25 letter alphabet

The results are shown in Table 2. The data shows a clear time/memory tradeoff for DNA sequences, gsufsort being faster while using approximately 1.25 more memory, gsufsort-light using slightly less memory then mkESA but taking more time. On proteins, gsufsort-light is only marginally slower than gsufsort but faster than mkESA. The authors of mkESA reported a 32% gain on a large protein dataset using 16 threads [17], but larger ${\mathsf {lcp}}$ values seem not to favor mkESA when compared to gsufsort-light, which is 47.9% faster on proteins and 12.9% faster on DNA.

**Table 2:** Algorithms’ running times and memory usage on different datasets collections

Collection	gsufsort			gsufsort-light			mkESA		
	Time	RAM	Bytes/N	Time	RAM	Bytes/N	Time	RAM	Bytes/N
shortreads	*4:25:52*	336.00	21.00	5:30:54	*272*.*00*	*17*.*00*	4:51:48	274.73	17.17
reads	*5:00:27*	336.00	21.00	5:10:04	*272*.*00*	*17*.*00*	5:44:58	280.68	17.54
pacbio	*4:19:37*	336.04	21.00	4:54:21	*272*.*03*	*17*.*00*	4:26:39	272.58	17.03
pacbio.1000	*4:28:22*	336.00	21.00	5:20:39	*272*.*00*	*17*.*00*	4:44:50	272.32	17.02
uniprot	*5:11:33*	336.90	21.00	5:25:37	*272*.*73*	*17*.*00*	9:58:03	294.86	18.38
gutenberg	*4:17:52*	334.40	21.00	4:53:05	*269*.*90*	*17*.*00*	–	–	–
random.dna	*4:23:56*	331.08	21.00	5:41:45	*268*.*02*	*17*.*00*	4:28:43	268.33	17.02
random.protein	5:20:06	331.08	21.00	5:47:38	*268*.*02*	*17*.*00*	*4:37:16*	268.33	17.02

Columns RAM and bytes/N show the peak memory in GB and the bytes per input symbol ratio. Each symbol of $T[0,N-1]$ uses 1 byte. Results for gutenberg are reported for gsufsort and gsufsort-light only, as mkESA is restricted to DNA and amino-acid alphabets. The best results are indicated in italics

The memory ratio (bytes/N) of gsufsort and gsufsort-light is constant, 21 and 17 bytes per input symbol respectively, corresponding to the space of the input string *T* (*N* bytes) plus the space for arrays ${{\mathsf {S}}}{{\mathsf {A}}}$ and $\mathsf {LCP}$ (8*N* bytes each) and, only for gsufsort, the space for ${{\mathsf {D}}}{{\mathsf {A}}}$ (4*N* bytes).

**Fig. 1:**
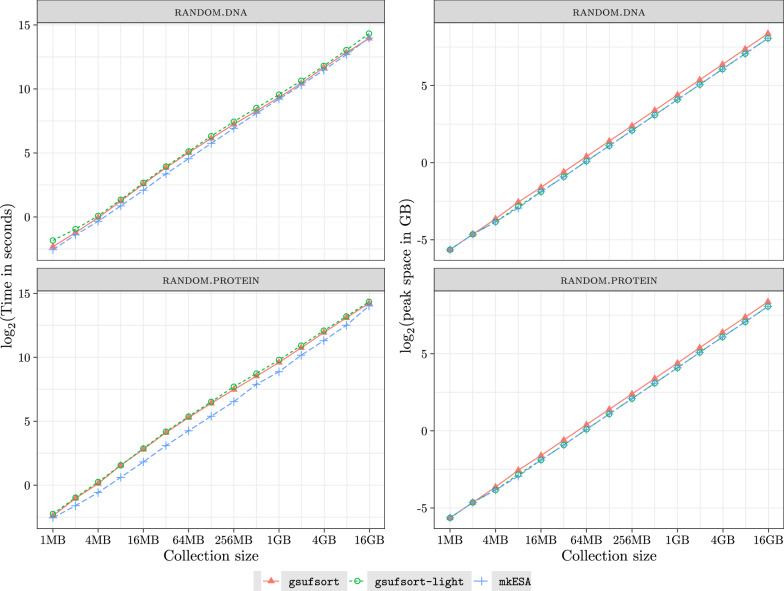
Running time in seconds and peak memory in GB (in logarithmic scale) on an random DNA and protein collections

We have also evaluated the performance of gsufsort, gsufsort-light and mkESA on collections of random DNA and random protein sequences. The collections have a growing number of 1MB sequences. The running time in seconds and the peak memory usage in GB are shown in Fig. 1 (logarithmic scale). Using random sequences reduces the variation due to ${\mathsf {lcp}}$ among collections. We can see a perfectly steady behavior of mkESA. While still *O*(*N*), gsufsort displays a deviation due to larger constants.

## Conclusions

We have introduced gsufsort, a fast, portable, and lightweight tool for constructing the suffix array and additional data structures for string collections. gsufsort may be used to pre-compute indexing structures and write them to disk, or may be included as a component in different applications. As an additional advantage, gsufsort is not restricted to biological sequences, as it can process collections of strings over ASCII alphabets.

## Availability and requirements

- Project name: gsufsort
- Project home page: http://www.github.com/felipelouza/gsufsort
- Operating system(s): Platform independent
- Programming language: ANSI C
- Other requirements: make, zlib (optional)
- License: GNU GPL v-3.0.

## Data Availability

The source code of the proposed algorithm is available at https://www.github.com/felipelouza/gsufsort.

## Footnotes

1 https://zlib.net

2 http://lh3lh3.users.sourceforge.net/kseq.shtml

3 http://www.bibiserv.cebitec.uni-bielefeld.de/mkesa
